# Clinical profile and short-term outcomes of acute myocardial infarction patients

**DOI:** 10.6026/973206300222966

**Published:** 2026-05-31

**Authors:** Reazun Nahar, Sakib Arsalan, Monija Islam Diba, Muhammad Ali Raza, Abdul Subhan Safdar, Manahil Zahra, Bushra Lail Shah

**Affiliations:** 1 Department of Care of Elderly, Medway Maritime Hospital, Gillingham, UK; 2 Department of Medicine, Medway Maritime Hospital, Gillingham, UK; 3 Department of Gastroenterology, DHQ Teaching Hospital, Gujranwala, Pakistan; 4 General Physician, General Medicine, Dawaa Tabuk Medical Complex, KSA; 5 Department of Medicine, Ayub Teaching Hospital, Abbottabad, Pakistan; 6 Department of Cardiology, Federal government polyclinic hospital, Islamabad, Pakistan

**Keywords:** Acute myocardial infarction, STEMI, risk factors, mortality, emergency care

## Abstract

Delayed recognition and poor early risk stratification of acute myocardial infarction remain major contributors to in-hospital complications and mortality. Therefore, it is of interest to evaluate clinical characteristics, risk factors and short-term outcomes in 200 patients presenting with acute myocardial infarction. The mean age was 56.8 ± 11.9 years, with STEMI accounting for 61% and hypertension present in 58% of patients. In-hospital complications occurred in 37% and mortality was 8%, significantly associated with higher Killip class, reduced LVEF, cardiogenic shock and delayed presentation. Thus, early risk stratification and timely management are essential to improve patient outcomes.

## Background:

Acute myocardial infarction (AMI) remains one of the most critical cardiovascular emergencies worldwide and continues to be a leading cause of morbidity and mortality despite major therapeutic advances. It represents the irreversible necrosis of myocardial tissue due to prolonged ischemia [1]. The burden of AMI is substantial not only in high-income countries but increasingly in low- and middle-income regions, where demographic transitions, urbanization and lifestyle changes have accelerated the prevalence of cardiovascular risk factors [2]. Patients presenting to the emergency department (ED) with AMI often show a broad and sometimes atypical clinical spectrum. While classic symptoms such as retrosternal chest pain radiating to the left arm or jaw remain common, a significant proportion of patients, particularly the elderly, women and individuals with diabetes mellitus, present with atypical features including dyspnea, epigastric discomfort, syncope, or unexplained fatigue [3]. Early recognition in the ED is crucial, as time-sensitive interventions, such as primary percutaneous coronary intervention or thrombolytic therapy, significantly reduce infarct size and improve survival [4]. The pathophysiology of AMI is closely linked to atherosclerotic coronary artery disease and is strongly influenced by modifiable and non- modifiable risk factors. Established risk factors include hypertension, diabetes mellitus, dyslipidemia, smoking, obesity, sedentary lifestyle and family history of premature coronary artery disease [5]. In recent years, additional contributors such as metabolic syndrome, chronic kidney disease, psychosocial stress and inflammatory states have been recognized as important determinants of cardiovascular risk [6]. The clustering of these factors significantly increases the likelihood of plaque instability and subsequent coronary thrombosis [7]. Therefore, it is of interest to evaluate the clinical profile, associated cardiovascular risk factors and short-term in-hospital outcomes of patients presenting to the emergency department with acute myocardial infarction and to identify predictors of early complications and mortality to support improved risk stratification and timely management.

## Methodology:

This was a hospital-based cross-sectional analytical study conducted at Sir Ganga Ram Hospital Lahore from January 2025 till July 2025. Acute myocardial infarction was diagnosed based on standard clinical criteria, including characteristic chest pain or equivalent ischemic symptoms, electrocardiographic changes suggestive of ST-elevation or non-ST-elevation myocardial infarction and elevated cardiac biomarkers (troponin I or T) in accordance with established international guidelines. Both ST-elevation myocardial infarction (STEMI) and non-ST-elevation myocardial infarction (NSTEMI) cases were included. Inclusion criteria comprised patients aged 18 years or older presenting to the emergency department with a confirmed diagnosis of AMI and who provided informed consent (or whose attendants provided consent, where applicable). Patients with unstable angina without biomarker elevation, those with prior recent myocardial infarction within the last 30 days and individuals with incomplete medical records were excluded from the study. Data were collected using a structured proforma. Demographic variables included age, gender and marital status. Clinical variables included presenting symptoms, duration of symptoms before hospital arrival, vital signs at presentation, Killip class and type of myocardial infarction (STEMI/NSTEMI). Cardiovascular risk factors assessed included hypertension, diabetes mellitus, dyslipidemia, smoking status, obesity (based on body mass index), family history of coronary artery disease and history of previous cardiovascular events. Laboratory investigations, electrocardiographic findings and echocardiographic parameters (including left ventricular ejection fraction) were recorded. Short-term outcomes assessed during hospitalization included arrhythmias, acute heart failure, cardiogenic shock, need for mechanical ventilation, requirement of inotropic support, length of hospital stay and in-hospital mortality. All patients were followed from admission until discharge or death. Data were entered and analyzed using Statistical Package for Social Sciences (SPSS) version 26. Continuous variables were expressed as mean ± standard deviation, while categorical variables were presented as frequencies and percentages. The independent sample t-test or MannWhitney U test was applied for comparison of continuous variables and the chi-square test or Fisher's exact test was used for categorical variables as appropriate. Logistic regression analysis was performed to identify independent predictors of adverse short-term outcomes. A p-value of <0.05 was considered statistically significant.

## Results:

A total of 200 patients presenting with acute myocardial infarction (AMI) were included. The mean age was 56.8 ± 11.9 years and 68% were male. STEMI was more common than NSTEMI (61% vs 39%). Chest pain was the predominant symptom (89%) and most patients presented in Killip class I (72%) (Table 1). The mean left ventricular ejection fraction (LVEF) was 45.7 ± 8.9%, with 28% having reduced LVEF (<40%).Hypertension (58%), diabetes mellitus (46%) and smoking (38%) were the most frequent risk factors and 63% had multiple (≥2) risk factors. In-hospital complications occurred in 37% of patients, with arrhythmias (18%) and acute heart failure (14%) being most common. The mean hospital stay was 4.9 ± 2.3 days. Overall in-hospital mortality was 8% and was significantly associated with higher Killip class,reduced LVEF, cardiogenic shock and delayed presentation (p < 0.05) (Table 2).

## Discussion:

This study evaluated the clinical profile, cardiovascular risk factors and short-term outcomes of patients presenting with acute myocardial infarction (AMI) to the emergency department. The findings demonstrate that AMI predominantly affected middle-aged to elderly males, with a high burden of modifiable cardiovascular risk factors. STEMI constituted the majority of cases and although most patients presented in Killip class I, a considerable proportion developed early in-hospital complications. The mean age of 56.8 years reflects the earlier onset of coronary artery disease frequently observed in developing regions, where cardiovascular events tend to occur nearly a decade earlier compared to Western populations. The predominance of male patients (68%) is consistent with established epidemiological patterns, although the growing incidence among females warrants attention [8]. Previous research has similarly reported male predominance and comparable age distributions among AMI cohorts presenting to emergency settings. Hypertension, diabetes mellitus and smoking were the most prevalent risk factors in this study, with nearly two-thirds of patients having multiple (≥2) risk factors. This clustering of modifiable factors significantly increases the likelihood of plaque instability and acute coronary thrombosis [9]. Previous research has demonstrated similar trends, highlighting hypertension and diabetes as leading contributors to acute coronary syndromes. The high prevalence of smoking further emphasizes the need for aggressive primary prevention strategies and community-level interventions [10, 11].

Chest pain was the dominant presenting symptom, reinforcing its diagnostic significance in emergency triage. However, the presence of dyspnea and other atypical symptoms in a subset of patients underscores the importance of maintaining a high index of suspicion, particularly in diabetics and elderly individuals. The predominance of STEMI cases in this cohort may reflect delayed healthcare access or limited pre-hospital screening, a pattern also observed in previous regional studies [12]. Short-term complications occurred in more than one-third of patients, with arrhythmias and acute heart failure being most frequent. The overall in-hospital mortality rate of 8% aligns with outcomes reported in similar hospital-based cohorts. Importantly, mortality was strongly associated with higher Killip class, reduced left ventricular ejection fraction (LVEF <40%), cardiogenic shock and delayed presentation [13]. These findings are consistent with established prognostic models, where hemodynamic instability and impaired ventricular function remain the strongest predictors of early mortality. Previous research has consistently demonstrated that Killip classification and LVEF are powerful determinants of short-term survival following AMI [14]. The association between delayed presentation and increased mortality highlights a critical gap in early recognition and timely reperfusion. Delays beyond the recommended therapeutic window substantially reduce the benefits of reperfusion therapy and increase infarct size. This finding reinforces the need for improved public awareness, efficient emergency referral systems and streamlined in-hospital protocols to minimize door-to-balloon time [15]. This study has several limitations that should be acknowledged. First, being a single-center study conducted in the emergency department setting, the findings may not be generalizable to other institutions or broader populations. Second, the cross-sectional design limits the ability to establish causal relationships between risk factors and short-term outcomes. Third, follow-up was restricted to in-hospital outcomes and long-term complications or post-discharge mortality was not assessed. Additionally, reliance on medical records and patient self-reporting for certain risk factors such as smoking may have introduced reporting bias. Finally, advanced biomarkers and detailed angiographic data were not uniformly available for all patients, which may have limited deeper risk stratification analysis.

## Conclusion:

We show that acute myocardial infarction patients presenting to the emergency department show a high burden of modifiable cardiovascular risk factors, with STEMI being the predominant presentation. Early clinical severity indicators such as higher Killip class, reduced left ventricular ejection fraction, cardiogenic shock and delayed hospital arrival were strongly associated with adverse short-term outcomes and in-hospital mortality.

## Advancement to knowledge:

This study provides updated local evidence on acute myocardial infarction presentation and early outcomes, identifying key predictors of in-hospital mortality that may improve emergency department triage and management.

## Figures and Tables

**Table 1:** Baseline clinical characteristics and risk factors (N = 200)

**Variable**	**Category**	**n (%) / Mean ± SD**
Age (years)	Mean ± SD	56.8 ± 11.9
Male Gender	-	136 (68.0%)
Type of MI	STEMI	122 (61.0%)
	NSTEMI	78 (39.0%)
Chest Pain	Present	178 (89.0%)
Killip Class I	-	144 (72.0%)
LVEF (%)	Mean ± SD	45.7 ± 8.9
LVEF <40%	-	56 (28.0%)
Hypertension	Present	116 (58.0%)
Diabetes Mellitus	Present	92 (46.0%)
Dyslipidemia	Present	82 (41.0%)
Smoking	Present	76 (38.0%)
Obesity	Present	68 (34.0%)
≥2 Risk Factors	Present	126 (63.0%)

**Table 2:** Short-term in-hospital outcomes and mortality predictors

**Variable**	**Category**	**n (%) / Mean ± SD**	**p-value**
Any Complication	Yes	74 (37.0%)	-
Arrhythmias	Yes	36 (18.0%)	-
Acute Heart Failure	Yes	28 (14.0%)	-
Cardiogenic Shock	Yes	12 (6.0%)	<0.001
Mechanical Ventilation	Required	18 (9.0%)	-
Inotropic Support	Required	22 (11.0%)	-
Length of Stay (days)	Mean ± SD	4.9 ± 2.3	-
In-Hospital Mortality	Yes	16 (8.0%)	-
Killip Class III-IV (among deaths)	-	62.50%	<0.001
LVEF <40% (among deaths)	-	75.00%	0.003
Delayed Presentation (>6 hrs)	-	56.30%	0.017
